# Enteric Fever in India

**Published:** 1889-12-14

**Authors:** 


### The Practitioner's Outlook and Retrospect.

[*The Editor will be glad to receive offers of co-operation and contributions from members of the profession. There is no doubt that much valuable material full of interest and instruction is at present lost to science. This is largely so in the case of* (1) *the younger and less-known members of the hospital staff,* (2) *those connected with cottage hospitals, and* (3) *the great body of medical practitioners not attached to any institution. The co-operation of all is cordially invited to enable the Editor to make* The Hospital *of the utmost possible use and value to the profession. Specialists willing to review books on their special subjects are invited to communicate this fact at once. All letters should be addressed to* The Editor, The Lodge, Porchester Square, London, W.]

### MEDICINE.

### ENTERIC FEVER IN INDIA.

In an article, published in a recent number of the *Indian Medical Gazette*, Deputy Surgeon-General J. B. Hamilton has some remarks on "enteric fever in India." He truly states that all enquiries into outbreaks of enteric fever in India are surrounded by greater difficulties than at home. For there are no sewers, drains, cisterns, traps, etc., defects in which are so often presumed to cause enteric fever in Europe. Dr. Hamilton, however, thinks that it may be traced to the milk supply in some instances, and to bazaar "pop" in others; and he recites instances tending to prove this. Dr. Hamilton also enters into the question of the prevalence or otherwise of enteric fever among natives of India, which he believes is not uncommon. As preventitive measures Dr. Hamilton mentions educating the masses on sanitary subjects, which, however, must take years, if not generations. Next he thinks an enactment against milk adulteration is required, and also against feeding milk cattle on litter or other improper food. Now, it is only in comparatively recent years that the term enteric or typhoid fever has appeared prominently in Indian mortality reports. But during the last quarter of a century or so, enteric has been attaining more and more notoriety as a cause of illness and death, particularly among young soldiers. Morehead, in his first "Researches on Indian Diseases," did not include typhoid fever as a disease of the country, although at a later date he admitted the occasional occurrence of the malady in India. There is, however, good reason to believe that enteric or typhoid fever has always prevailed in that country. It must be recollected that it is only comparatively recently that fevers have been differentiated in Europe into the varieties and classes as they now stand in the nomenclature of disease. Enteric fever is supposed to be distinguished by a peculiar rash and by a peculiar bowel complaint. The rash, however, on the dark skin of the native of India may easily be overlooked, or if seen it may be regarded as flea-bite, and doubtless was so regarded years back when special attention had not been directed to the subject. Then all kinds of fevers in India are often attended with diarrhœa, a malady in itself so common in the Eastern tropics. If older authors were referred to, such as Allan Webb, Annesly, Johnson, etc., it would be seen that they describe diseases under the term remittent fever and otherwise which would now in all probability be classed as enteric. Many of the fevers of India are, however, so unlike any particular type that they have been termed "hybrid"; or they have been regarded as malarious modified by typhoid influences; or as typhoid modified by malarious influences. Hence we have such terms introduced as "typho-malarial," "malario-typhoid," "typho-remittent," etc. These fevers, while not presenting all the symptoms of any type, are yet the blurred image of all, and able physicians diagnose them differently, and even the same physician would probably make a different diagnosis on a different day. Sir J. Fayrer uses the term "climatic fever" as the one most applicable to such cases, and Sir William Moore terms these hybrid fevers "undefined climate fevers," and this author remarks, "A considerable proportion, if not the great majority, of the fevers of India seem a mixture of several phases, based on a few prominent symptoms common to all. Thus the disease may sometimes most resemble remittent, at others typhus, typhoid, or relapsing; yet not be found fairly distinguishable as either according to the dicta enunciated by the exponents of fever types, but yet it may be the blurred image of all." Lastly, it may be remarked that the causes of enteric in this country are not the causes in India, but it is, as Sir J. Fayrer remarks, quite probable that the cause of enteric is not limited to one agency.

